# Olaquindox disrupts tight junction integrity and cytoskeleton architecture in mouse Sertoli cells

**DOI:** 10.18632/oncotarget.20289

**Published:** 2017-08-16

**Authors:** Di Wu, Chun-Jie Huang, Xiao-Fei Jiao, Zhi-Ming Ding, Jia-Yu Zhang, Fan Chen, Yong-Sheng Wang, Xiang Li, Li-Jun Huo

**Affiliations:** ^1^ Key Laboratory of Agricultural Animal Genetics, Breeding and Reproduction of Ministry of Education, College of Animal Science and Technology, Huazhong Agricultural University, Wuhan 430070, Hubei, China; ^2^ Department of Hubei Province Engineering Research Center in Buffalo Breeding and Products, Wuhan 430070, Hubei, China

**Keywords:** Sertoli cell, olaquindox, tight junction, cytoskeleton, blood-testis barrier

## Abstract

Sertoli cells, by creating an immune-privileged and nutrition supporting environment, maintain mammalian spermatogenesis and thereby holds the heart of male fertility. Olaquindox, an effective feed additive in livestock industry, could potentially expose human into the risk of biological hazards due to its genotoxicity and cytotoxicity, highlighting the significance of determining its bio-safety regarding human reproduction. Herein, we deciphered the detrimental effects of olaquindox on male fertility by mechanistically unraveling how olaquindox intervenes blood-testis barrier in mouse. Olaquindox (400 μg/ml) exposure significantly compromised tight junction permeability function, decreased or dislocated the junction proteins (e.g., ZO-1, occludin and N-cadherin) and attenuated mTORC2 signaling pathway in primary Sertoli cells. Furthermore, olaquindox disrupted F-actin architecture through interfering with the expression of actin branching protein complex (CDC42-N-WASP-Arp3) and actin bunding protein palladin. Olaquindox also triggered severely DNA damage and apoptosis while inhibiting autophagic flux in Sertoli cell presumably due to the exacerbated generation of reactive oxygen species (ROS). Pre-treatment with antioxidant N-acetylcysteine effectively ameliorated olaquindox-induced exhaustion of ZO-1 and N-Cadherin proteins, DNA damage and apoptosis. More significantly, olaquindox disrupted the epigenetic status in Sertoli cells with hypermethylation and concomitantly hypoacetylation of H3K9 and H3K27. Overall, our study determines olaquindox targets Sertoli cells to affect BTB function through tight junction proteins and F-actin orgnization, which might disrupt the process of spermatogenesis.

## INTRODUCTION

During spermatogenesis, Sertoli cells offer nutritional and physical support to the developing germ cells, and creates an immuno-privileged microenvironment by blood-testis barrier (BTB) spatially sequestering germ cells from autoimmunity and ectogenesis stimulus and thereby, fostering the completion of meiosis [1, 2]. BTB which is composed of the tight junction (TJ) together with basal ectoplasmic specialization (basal ES) and gap junction (GJ) by the adjacent Sertoli cells undergoes periodically reconstruction to facilitate the transition of preleptotene spermatocytes from basal to apical compartment during seminiferous epithelial cycle promoted by cytokines and chemicals [3, 4]. BTB catastrophe has been recognized as an important contributor for the environmental endocrine-disrupting compounds (EDCs) and other chemicals-induced dysfunction of spermatogenesis [5–7]. The damaging effects of these toxicants to testicular function are mediated by mitogen-activated protein kinases downstream, which in turn perturbs the actin bundling and accelerates the actin-branching activity, result in impaired spermatogenesis [8, 9]. In addition, oxidative stress-caused by inflammatory cytokines and ectogenesis stimulus induced TJ barrier disruption [10–13].

Olaquindox (OLA, 2-(*N*-2-hydroxyethyl-carbamonyl)-3-methyl-quinoxaline-N^1^, N^4^-dioxide), a common medicinal feed additive in livestock husbandry for growth promoting and antibacterial purpose to prevent dysentery and bacterial enteritis, has been banned for using by the European Commission of the European Community and Canada since 1998 due to its potential risks such as genotoxic, mutagenic and photoallergenic effects [14–16]. Notably, it is still being widely used in China due to its beneficial effects, which potentially exposes our human, if consume those animal products with residual OLA due to its illogical usage, into the risks of its biological hazards. Recent studies have demonstrated that a relatively low concentration of olaquindox significantly induced DNA mutation, and the frequency of which was increased by up to 12-fold [17], and the cumulative toxicity of olaquindox has also been reported [18–20]. More importantly, OLA could incite DNA damage and apoptosis [21–23]. Despite OLA could be categorized as mutagenic and carcinogenic with developmental and reproductive toxicities [24] whether OLA has deleterious effects on mammalian spermatogenesis, or more specifically, the BTB dynamic and if so, what the underlying mechanisms might be involved in still remain to be explored.

In this study, we in depth determine that OLA perturbs the TJ permeability function by attenuating the cell-cell interface distribution of the tight junction proteins and the architecture of F-actin. Moreover, OLA potentiates DNA damage and apoptosis due to aggravation of ROS production, and disrupts the epigenetic status in Sertoli cells. Overall, our study pinpoint that olaquindox targets Sertoli cells to affect BTB function, which will help us to develop a better appreciation of the toxicological feature of OLA in male reproductive biology.

## RESULTS

### OLA compromises Sertoli cells survival

**Figure 1:**
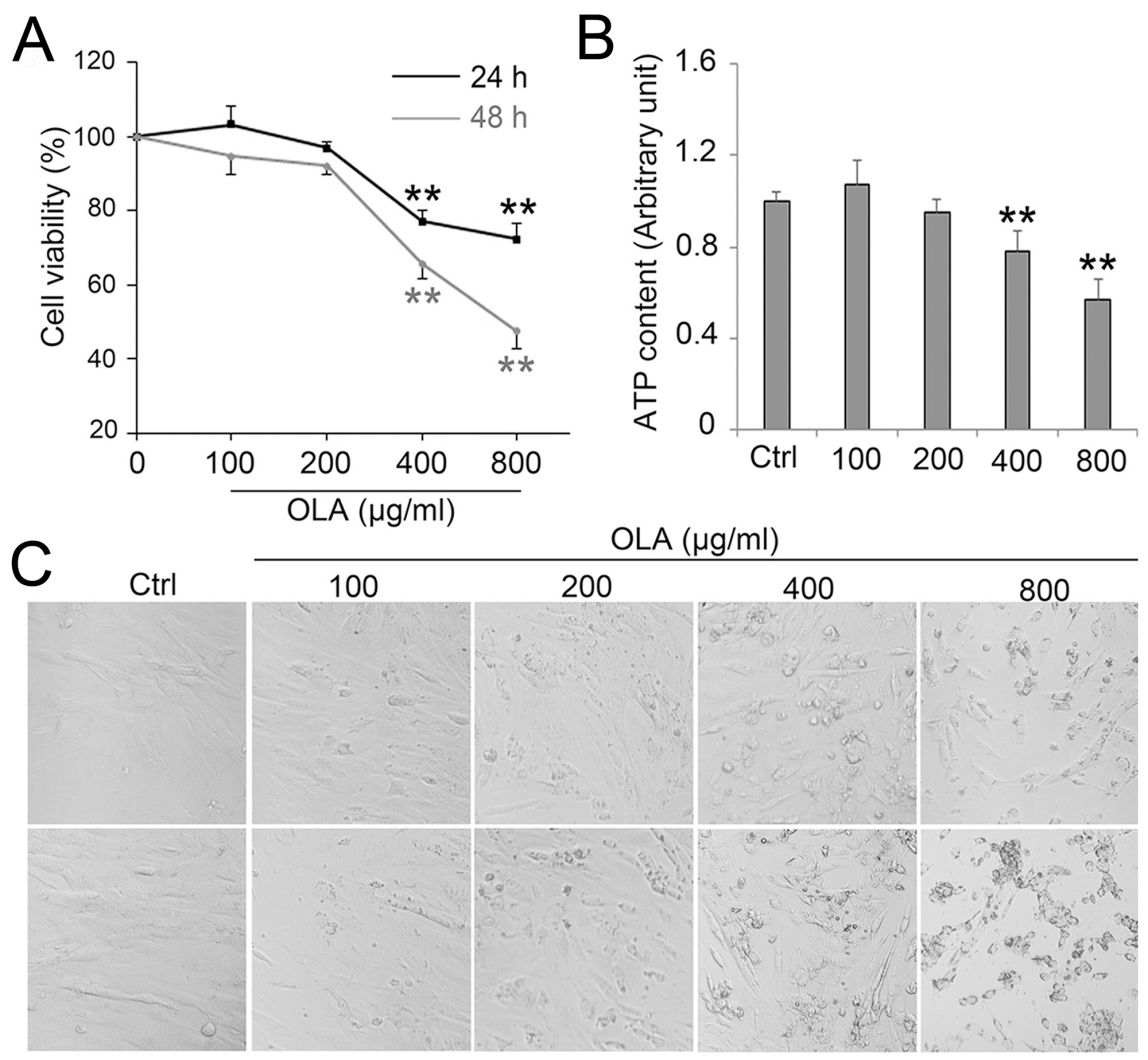
OLA decreased Sertoli cell viability **(A)** Cell viability was determined by CCK-8 assay in Sertoli cells treated with the indicated concentrations of OLA for 24 h and 48 h. Cells treated with vehicle (0.2% DMSO) were used as negative control (0). Date are presented as mean ± S.E.M. (three independent replicates per groups). * p < 0.05; ** p < 0.01. **(B)** Content of ATP was measured by luciferase assay followed the indicated concentrations of OLA for 24 h. Cells treated with vehicle (0.1% DMSO) were used as negative control. Date are presented as mean ± S.E.M. (three independent replicates per groups). * *p* < 0.05; ** *p* < 0.01. **(C)** Cell morphology were observed using an inverted microscope (Nikon, Japan).

To investigate the effect of OLA on Sertoli cells viability, CCK-8 assay was performed after Sertoli cells treated with a concentration gradient of OLA for 24 h and 48 h. The cell viability was significantly decreased along with the increase of OLA concentration after 24 h and 48 h treatment, respectively, indicating that OLA effectively inhibited cell viability in a dose-dependent manner (Figure 1A). The abundance of ATP, which is the product of cellular energy metabolism in metabolically active cells, also experienced a conspicuous decline after 24 h treatment with 400- or 800 μg/ml OLA (Figure 1B). Moreover, exposure with different concentrations of OLA for 24 h and 48 h significantly led to a reduction in the number of living cells and morphology changes with shrinking, rounding, cell vacuolization and detachment morphologies in Sertoli cells (Figure 1C), further confirming that OLA exposure compromises Sertoli cells survival.

### OLA disrupts the TJ permeability barrier

**Figure 2:**
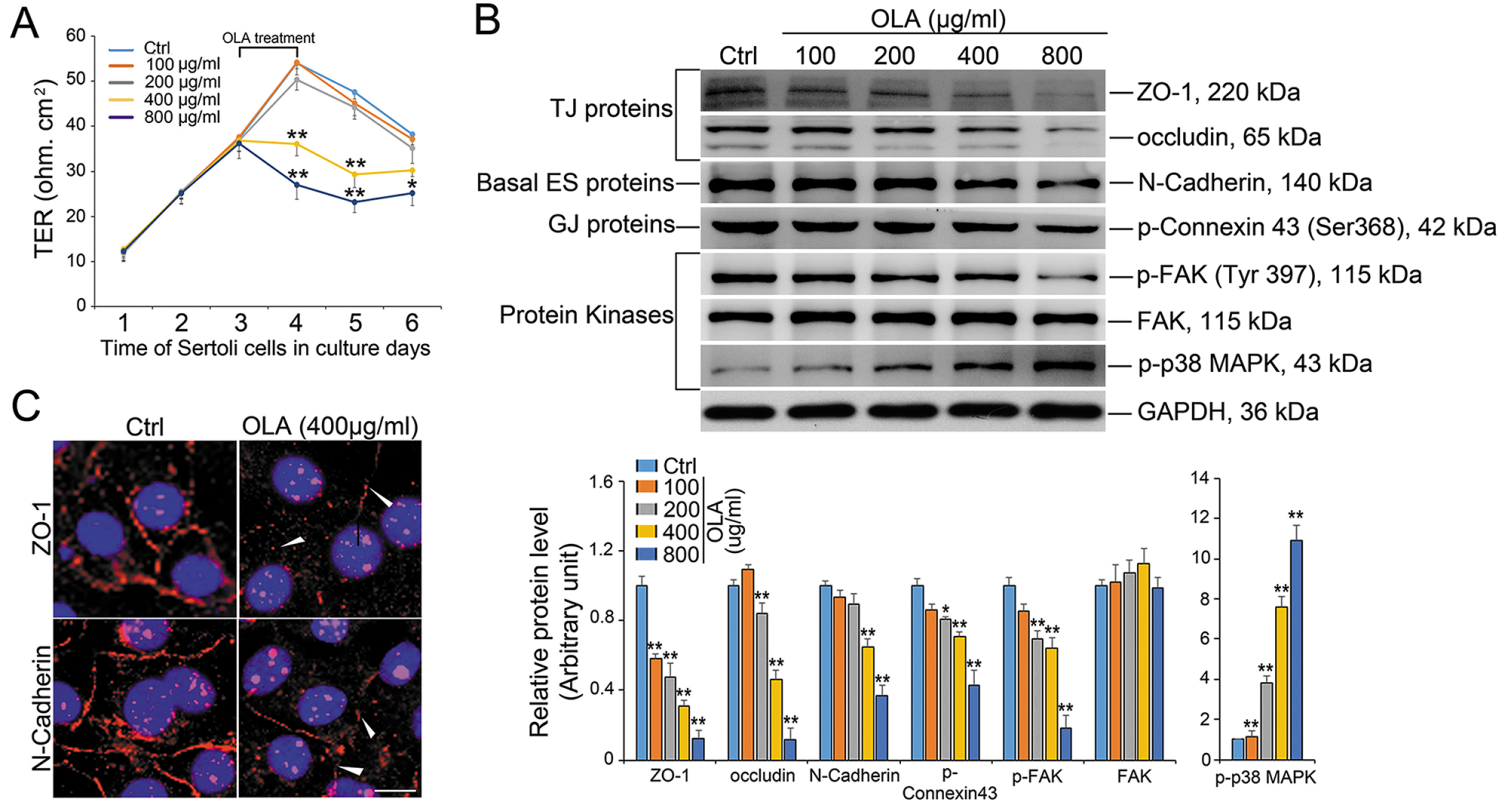
OLA perturbs Sertoli cell BTB function Sertoli cells cultured on Matrigel-coated bicameral units, dishes or coverslips were treated on day 3 with 100-, 200-, 400- or 800 μg/ml of OLA for 24 h. Thereafter, cells were washed twice with PBS to remove residual OLA and terminated for immunoblot (IB) or IF on day 4. Cells treated with vehicle (0.2% DMSO) were used as negative control. **(A)** Graph showing the integrity of TJ permeability barrier after OLA *vs*. vehicle control (0.2% DMSO) treatment. Date are presented as mean ± S.E.M. (three independent replicates per groups). * *p* < 0.05; ** *p* < 0.01. **(B)** Immunoblot analysis to assess the effects of OLA on the expression of TJ proteins: ZO-1 and occludin; basal ES proteins: N-cadherin; protein kinases: FAK, p-FAK and p-p38MAPK. GAPDH served as protein loading control. Semiquantitative analysis of protein expression in following histogram (mean ± S.E.M., three independent replicates per groups). * *p* < 0.05; ** *p* < 0.01. **(C)** Immunofluorescence analysis to assess the effects of OLA at 400 μg/ml *vs*. control on the distribution of TJ proteins: ZO-1 and basal ES proteins: N-cadherin. OLA caused internalization of TJ and basal ES proteins in Sertoli cells in which these proteins no longer tightly localized at the Sertoli cell-cell interface. ZO-1 and N-cadherin at the cell-cell interface were diminished following OLA treatment (white arrowheads). Nuclei were visualized by DAPI (blue). Scale bar, 20μm.

Sertoli cells cultured *in vitro* in ∼2-3 d are known to establish a functional permeability barrier that mimic the BTB *in vivo*, and this modle has been widely used by investigators in this field to study Sertoli cell TJ barrier function [3, 25]. To uncover whether OLA could influence BTB integrity, Sertoli cells were cultured for 3 d and treated with a concentration gradient of OLA for 24 h followed by determination of trans-epithelial electrical resistance (TER). Of note, OLA was found to induce a disruption of the TJ barrier function evidenced by the precipitous decrease in TER starting from the concentration of 400 μg/ml (Figure 2A). The BTB-associated proteins were then examined to confirm the dysfunction of BTB integrity. Significantly, the TJ proteins [e.g., zonula occludens 1 (ZO-1), occludin], basal ES protein (e.g., N-cadherin) and GJ protein (p-Connexin 43) were down-regulated. Additionally, the expression of the TJ regulator FAK (focal adhesion kinase) remains unchanged while its phosphorylated form (p-FAK^Y397^) was declined following OLA treatment. It was notable that p-p38 MAPK was also activated in a dose-dependent manner (Figure 2B). After OLA treatment (400 μg/ml), the weakened expression and mis-localization of TJ protein ZO-1 and basal ES protein N-cadherin at the Sertoli cell-cell interface were also noted wherein these proteins no longer tightly associated with the cell cortical zone, redistributing from the cell-cell interface to the cell cytosol (Figure 2C, white arrow). Taken together, we conclude that OLA could disrupt BTB integrity by interfering with the dynamics of the TJ and basal ES proteins at the BTB.

### OLA perturbs Sertoli cell cytoskeleton

**Figure 3:**
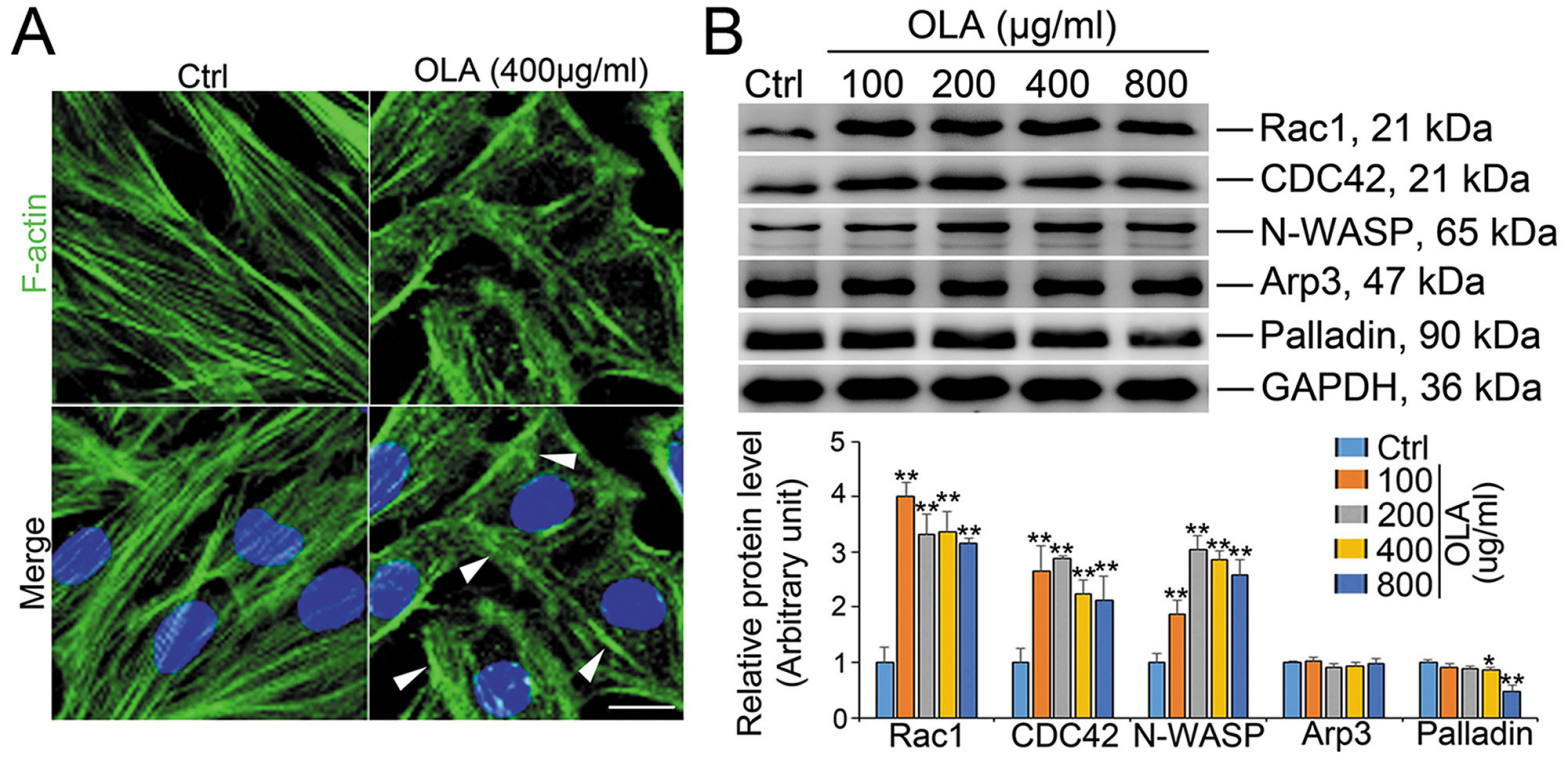
OLA disrupts actin microfilaments organization in Sertoli cells **(A)** Immunofluorescence analysis to assess the effects of OLA on the organization of actin microfilaments in Sertoli cells. Sertoli cells cultured for 2 d were treated with 400 μg/ml OLA or control (0.1% DMSO) for 24 h. Cells were fixed and processed for FITC-phalloidin (F-actin, green) and DAPI (nuclei, blue) staining. Significantly, OLA was prominently disorganized actin microfilaments with dense clusters (annotated by white asterisk). Scale bar, 20 μm. **(B)** Immunoblot analysis to assess the effects of OLA on the expression of actin regulatory proteins: Rac1, CDC42, N-WASP, Arp3 and Palladin. GAPDH served as protein loading control. Semiquantitative analysis of protein expression in following histogram (mean ± S.E.M., three independent replicates per groups). * *p* < 0.05; ** *p* < 0.01.

The regulation of actin dynamics in unique testicular junctions is crucial to spermatogenesis [26–28]. To pinpoint whether destruction of TJ barrier function in OLA-treated Sertoli cells could be ascribe to disrupted F-actin architecture, F-actin organization was therefore analyzed. As expected, F-actin architecture in Sertoli cells was significantly affected by 400 μg/ml OLA exposure with actin microfilaments highly aggregated at the cell periphery (white arrow) and few stretches fibers running across the cells (Figure 3A), implicating that the dis-organization of F-actin is likely to be the causative of the disrupted TJ function. To deeply uncover the underlying mechanism by which OLA dictates F-actin dynamics, the expression of several actin regulating proteins were then examined. As shown in Figure 3B, the abundance of N-WASP, the regulator of actin polymerization and activator of Arp2/3 complex, was increased while the branched actin polymerization protein Arp3 remains unchanged. The small GTPases of Rho family including Rac1 and CDC42, which act as the upstream activators of N-WASP, are the major regulators of actin cytoskeleton [29, 30]. Noteworthly, the expression of both Rac1 and CDC42 were abruptly elevated following OLA treatment starting from the dosage of 100 μg/ml. Palladin, a cytoskeletal scaffolding molecule that promotes actin bundling, associates with the actin filaments in Sertoli cells and thereby maintains the TJ function and spermatid transport [31]. Intriguingly, the abundance of palladin was decreased by a relative high concentration (400 μg/ml) of OLA exposure (Figure 3B). Overall, our results indicated that OLA disrupts F-actin architecture which consequently triggers the loss of cell polarity in Sertoli cells.

### OLA attenuates mTORC2 complex activity

**Figure 4:**
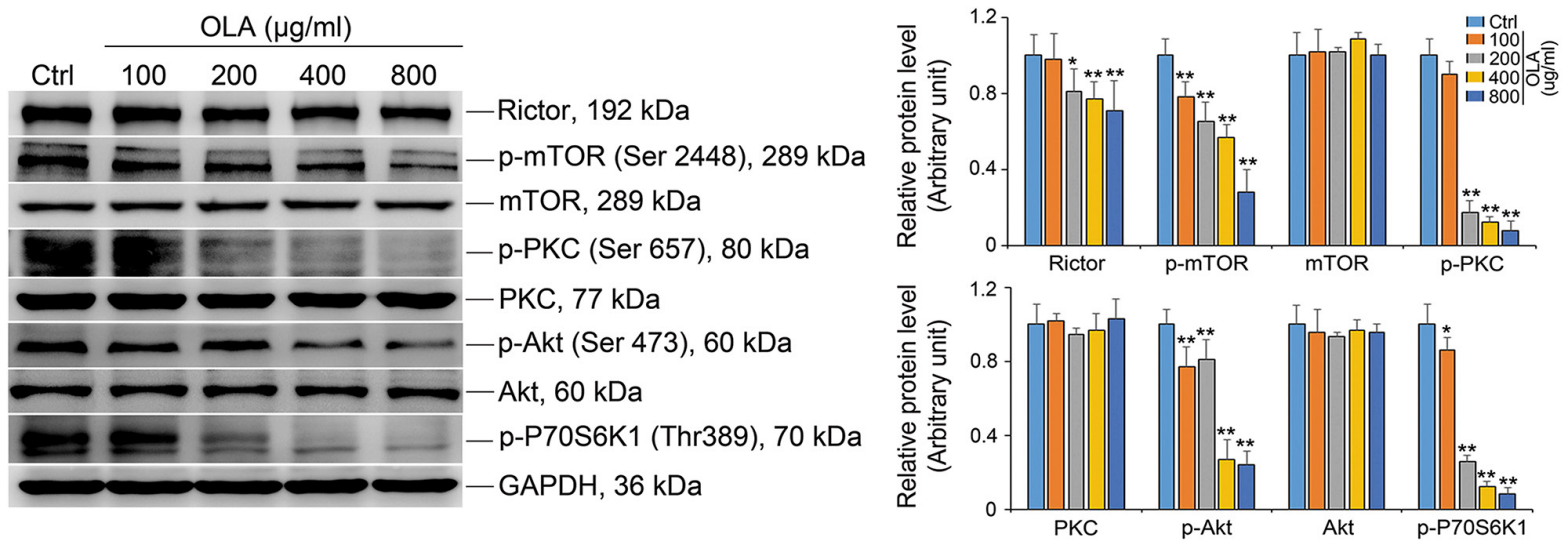
OLA attenuates mTORC2 complex activity Sertoli cells cultured on dishes were treated on day 3 with 100-, 200-, 400- or 800 μg/ml OLA for 24 h. Cells treated with vehicle (0.2% DMSO) were used as negative control. Thereafter, cells were washed twice with PBS to remove residual OLA and terminated for immunoblot (IB). Immunoblot analysis to assess the effects of OLA on the expression of mTORC2 complex: Ritor and p-mTOR, downstream proteins: PKC, Akt, p-PKC and p-Akt^Ser473^. GAPDH served as protein loading control. Semiquantitative analysis of protein expression in following histogram (mean ± S.E.M., three independent replicates per groups). * *p* < 0.05; ** *p* < 0.01.

The mammalian target of rapamycin (mTOR) is a well known non-receptor protein Ser/Thr kinase that orhestrate a spectrum of cellular biological events including cytoskeleton remodeling to assist BTB reconstruction during the epithelial cycle of spermatogenesis [32–36]. As such, we next examined the mTOR and rictor, which together with other binding partners form the mammalian target of rapamycin (mTOR) complex 2 (mTORC2) to regulate blood-testis barrier dynamics via effecting gap junction communications and actin cytoskeleton [34, 37]. As shown in Figure 4, rictor as well as the phosphorylated form of mTOR (p-mTOR^S2481^) were significantly decreased by OLA exposure in a dose dependent manner, implying that the level of functional mTORC2 was reduced following OLA treatment (Figure 4). mTORC2 has been implicated in regulation of BTB dynamics via PKCα and/or AKT pathway [37]. For this, we further determined the proteins in PKCα and AKT pathways by immunoblot analysis. As anticipated, the abundance of p-PKCα and p-AKT^Ser 473^ were indeed reduced regardless of the total levels of PKC-α and AKT remained unaltered. mTOR plays a critical role in governing cell proliferation by interfering with several translational effectors including p70S6 kinase [38] and the level of p-p70S6 kinase was obviously reduced in a OLA-dose dependent manner (Figure 4), confirming that the cell proliferation activity was dampened.

### OLA induces ROS production

**Figure 5:**
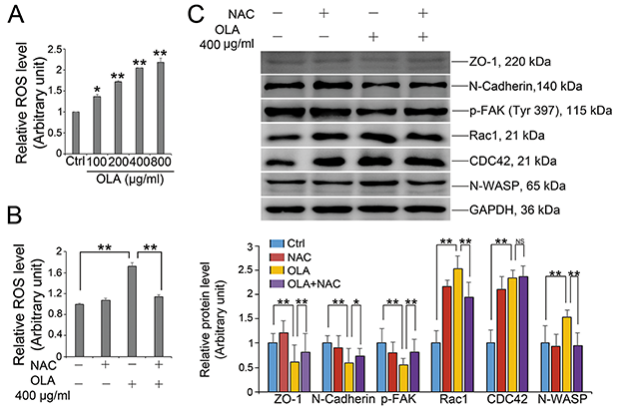
OLA induces ROS production **(A)** The level of ROS was measured by ROS-Glo™ H_2_O_2_ Assay Kit, after Sertoli cells were treated with the indicated concentrations of OLA for 24 h. Cells treated with vehicle (0.2% DMSO) were used as negative control. Date are presented as mean ± S.E.M. (three independent replicates per groups). * *p* < 0.05; ** *p* < 0.01. **(B)** Sertoli cells were pre-treated with NAC (10 mM) for 1 h, followed by OLA (400 μg/ml) treatment for 24 h prior to ROS assay. Date are presented as mean ± S.E.M. (three independent replicates per groups). * *p* < 0.05; ** *p* < 0.01. **(C)** Sertoli cells were pretreated with NAC(10 mM) for 1 h, followed by 400 μg/ml OLA treatment for 24 h prior to immunblot analysis for the expression of TJ protein (ZO-1), basal ES protein (N-Cadherin), p-FAK and actin regulator proteins (Rac1, CDC42, and N-WASP). GAPDH served as protein loading control. Semiquantitative analysis of protein expression in following histogram (mean ± S.E.M., three independent replicates per groups). * *p* < 0.05; ** *p* < 0.01, NS: not significant.

Oxidative stress represents as another common trigger of multiple chemicals-induced barrier disruption [10–12]. To reveal the effect of OLA on ROS production, Sertoli cells were treated with various concentrations of OLA for 24 h followed by determination of ROS generation with ROS-Glo™ H_2_O_2_ Assay Kit. OLA strikingly aggravated the cellular reactive oxygen species (ROS) content in a dose-dependent manner (Figure 5A). Pretreatment with N-acetylcysteine (NAC), a thiol antioxidant, effectively ameliorated the OLA exposure induced ROS production in Sertoli cells (Figure 5B). More importantly, pretreatment with NAC could also effectively alleviate the dysfunction of protein expression regarding BTB integrity (ZO-1, N-Cadherin and p-FAK) and actin regulation (Rac1 and N-WASP) (Figure 5C). Overall, the results indicated that ROS stress might be an important contributor to the compromised permeability function by interfering with the expression of BTB integrity proteins following OLA exposure.

### OLA causes DNA damage

**Figure 6:**
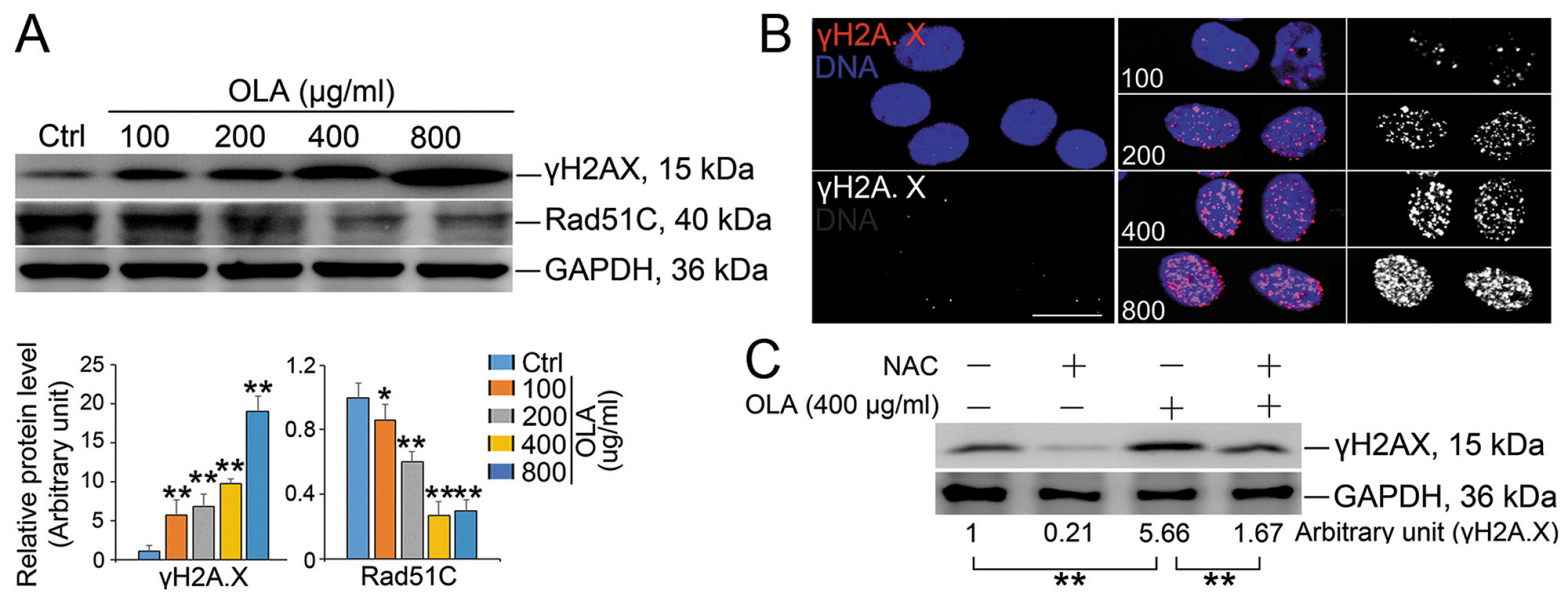
OLA induces DNA damage **(A)** Sertoli cells were treated with the indicated concentrations of OLA for 24 h, and treated with vehicle (0.2% DMSO) were used as negative control followed by immunblot analysis for the expression of γH2A.X and Rad51C. Semiquantitative analyses of protein expression in following histogram (mean ± S.E.M., three independent replicates per groups). * *p* < 0.05; ** *p* < 0.01. **(B)** Representative images showing the immunofluorescence staining of γH2A.X (red) and DAPI (nuclei, blue) in Sertoli cells after treatment with the indicated concentrations of OLA for 24 h. Scale bar, 20μm. **(C)** Sertoli cells were pretreated with NAC (10 mM) for 1 h, followed by 400 μg/ml OLA treatment for 24 h prior to immunblot analysis for the expression of γH2A.X. GAPDH served as protein loading control. Semiquantitative analysis of protein expression in following number (mean ± S.E.M., three independent replicates per groups). * *p* < 0.05; ** *p* < 0.01.

Even though intracellular ROS is required for many cellular functions and mild oxidative stress facilitates cell survival and growth adaptation, severe oxidative stress could cause DNA damage, cell senescence and even cell death [39]. Indeed, the potentiated DNA damage, which was revealed by the increased expression of γH2A.X, was obviously observed in OLA-exposed Sertoli cells (Figure 6A), which was further confirmed by the conspicuous increased in the γH2A.X foci (Figure 6B). Pretreatment with NAC, significantly, reduced the expression of γH2A.X to a lesser extent in OLA-exposed Sertoli cells implies that, in OLA-exposed Sertoli cells, the DNA damage might be largely caused by ROS stress (Figure 6C). In response to DNA damage, cells must timely and properly activate the related signaling cascades to modulate chromatin structure and organization within the DNA lesion region and thereby, creating a permissive environment for recruiting DNA damage repair (DDR) factors [40]. To examine whether the potentiated DNA damage in OLA-exposed Sertoli cells could be attributed to the dysfunction of DNA damage repairing event, the homologous recombination (HR) DNA repairing factor RAD51C was analyzed. Significantly, the expression RAD51C was gradually decreased following OLA treatment (Figure 6A). These results indicated that the DNA damage caused by ROS stress in OLA-exposed Sertoli cells was ascribed to the attenuation of DNA repairing.

### OLA inhibits autophagic flux and induces apoptosis in Sertoli cells

**Figure 7:**
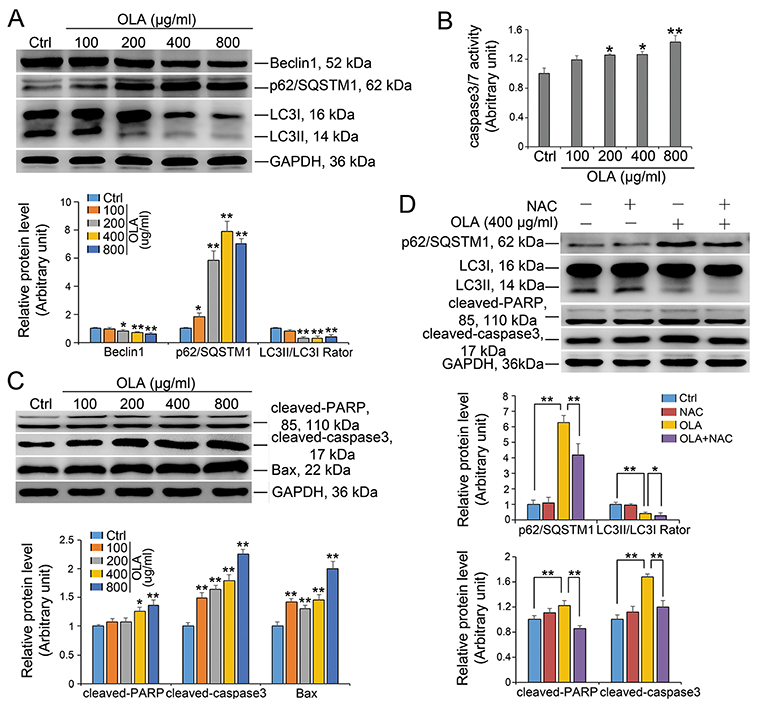
OLA inhibits autophagic flux and induces apoptosis Sertoli cells cultured on dishes were treated on day 3 with 100-, 200-, 400- or 800 μg/ml OLA for 24 h. Cells treated with vehicle (0.2% DMSO) were used as negative control. **(A)** Immunoblot analysis to assess the expression of autophagic flux. GAPDH served as protein loading control. Semiquantitative analyses of protein expression in following histogram (mean ± S.E.M., three independent replicates per groups). * *p* < 0.05, ** *p* < 0.01. **(B)** Caspase-3/7 activity was examined using the Caspase-3/7 assay Kit (Promega) according to the manufacturer’s protocol. Date are presented as mean ± S.E.M. (three independent replicates per groups). * *p* < 0.05; ** *p* < 0.01; NS: not significant. **(C)** Immunoblot analysis to assess the expression of cleaved-PARP, cleaved-caspase3 and Bax. Semiquantitative analyses of protein expression in following histogram (mean ± S.E.M., three independent replicates per groups). * *p* < 0.05; ** *p* < 0.01. **(D)** Sertoli cells were pretreated with NAC (10 mM) for 1 h, followed by 400 μg/ml OLA treatment for 24 h prior to immunblot analysis for the expression of each protein. Semiquantitative analysis of protein expression in following histogram (mean ± S.E.M., three independent replicates per groups). * *p* < 0.05; ** *p* < 0.01.

Autophagy and apoptosis are two main interlinked mechanisms for cells to eliminate the unwanted cellular materials and to determine the cell fate upon intrinsic and extrinsic stress insult [41, 42]. Autophagy in Sertoli cells was further assessed by examining autophagic flux, which is the dynamic process of autophagy. Intriguingly, Beclin-1 which is a critical factor that initiates autophagy was somehow significantly decreased in OLA-exposed Sertoli cells in a dose dependent manner (Figure 7A). Autophagic flux can be measured by the level of SQSTM1/p62 and LC3-II. The accumulation of SQSTM1/p62 and the strikingly reduction in LC3II/I ratio indicating that a weakened autophagic flux occurred in OLA treated Sertoli cells (Figure 7A). Pretreatment with NAC markedly inhibited the OLA exposure induced accumulation of SQSTM1/p62 (Figure 7D). When DNA damage cannot be efficiently repaired, cells will ultimately undergo necrosis and programmed cell death or namely apoptosis [42]. As shown in Figure 7B, the apoptosis, which was revealed by the caspase-3/7 activity, was steeply induced by OLA exposure in a dose-dependent manner. The increased expression of cleaved-PARP, cleaved-caspase 3 and Bax, which are well-recognized apoptosis markers, further confirmed the occurrence of apoptosis in OLA treated Sertoli cells (Figure 7C). Notably, the OLA induced apoptosis in Sertoli cells could be efficiently rescued by NAC pretreatment evidenced from the decline in the expression of cleaved-caspase 3 and cleaved-PARP (Figure 7D). Taken together, these results potently indicated that OLA induces apoptosis while inhibit autophagy in Sertoli cells.

### OLA causes histone modification change

**Figure 8:**
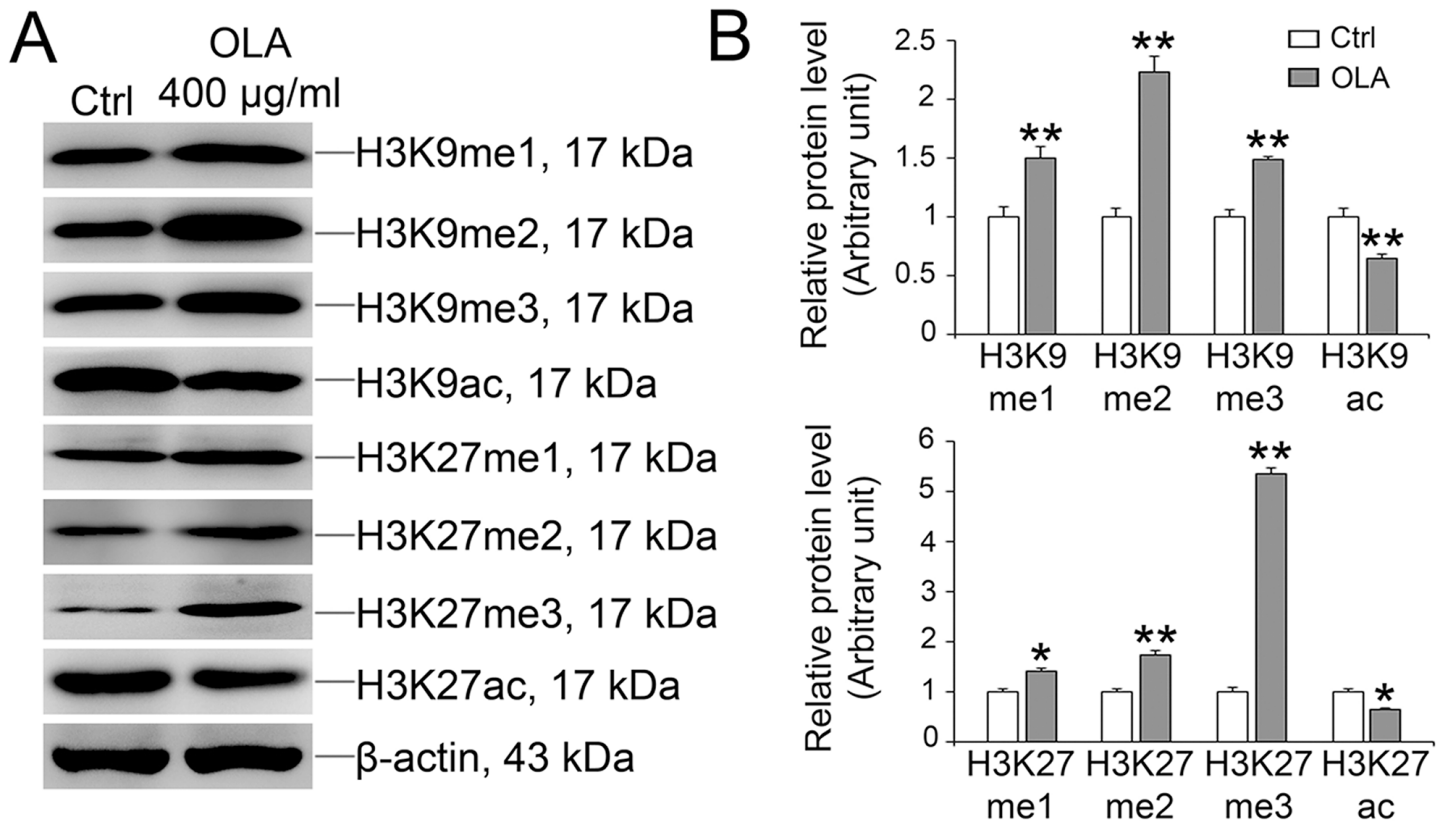
OLA increases the methylation and decreases acetylation of H3K9 and H3K27 Sertoli cells cultured on dishes were treated on day 3 with 400 μg/ml OLA for 24 h. Cells treated with vehicle (0.1% DMSO) were used as negative control. Thereafter, cells were washed twice with PBS to remove residual OLA and terminated for immunoblot (IB). **(A)** Immunoblot analysis to assess the effects of OLA on the expression of H3K9 and H3K27 modification. β-actin served as protein loading control. **(B)** Semiquantitative analysis of protein expression in following histogram (mean ± S.E.M., three independent replicates per groups). * *p* < 0.05; ** *p* < 0.01.

Histone modifications, such as methylation and acetylation, are critical for DNA damage repair and genome integrity [43–45]. Tri-methylation of histone H3 (H3K9me3) is locally and transiently up-regulated while the acetylation of H3 (H3K9ac) is down-regulated in the vicinity of DNA lesions [46, 47]. Of note, in OLA-treated (400 μg/ml) Sertoli cells, the methylation of H3K9 and H3K27, which mainly involves in regulating transcriptional activity and heterochromatic genome integrity was significantly augmented while the acetylation of H3K9 and H3K27, which are transcriptionally active chromatin marks [48], was concomitantly reduced (Figure 8). Notably, after NAC pretreatment, the hypoacetylation of H3K9 and H3K27 induced by OLA exposure could be efficiently rescued while the hypermethylation of H3K9 and H3K27 induced by OLA exposure was intriguingly somehow boosted (Supplementary Figure 1).

## DISCUSSION

Spermatogenesis, which is largely dictated by environmental, endocrine, paracrine and metabolic cues, produces the male germ cells or termed sperms and thereby determines the reproductive potential of males. Sertoli cells play a crucial role during spermatogenesis by offering nutritional and physical support and creating an immune-privileged environment to protect germ cells from endogenous and exogenous insults [1, 2]. Unfortunately, Sertoli cells usually are the target of multiple reproductive toxicants which compromise the spermatogenesis and the consequent fertility in male [49]. We herein for the first time deciphered that OLA has deleterious effects on male reproduction by interfering with the BTB integrity, cytoskeleton architecture, DNA damage and even histone epigenetic status in Sertoli cells. More importantly, our study could broaden our knowledge of the biological hazards of OLA to human health, which might help to develop a better appreciation of the regulation of OLA usage and environmental contamination.

Primary Sertoli cells after 2-3 days of culturing are known to form a functional and structurally ultra-structures that mimics the BTB *in vivo* [3, 4]. In this context, we use *in vitro* model to determine the underlying mechanisms by which OLA induces reproductive toxicity. We found that OLA possess cytotoxicity to Sertoli cell by reducing cell viability and ATP production. Moreover, OLA increases the tight junction permeability accompanied by a decline in trans-epithelial electrical resistance in Sertoli cells. TJ proteins (e.g., ZO-1 and occludin), basal ES protein (e.g., N-Cadherin) and GJ protein (e.g., connexin 43) are important structural components in Sertoli cells, which are essential for the regulation of BTB function [3]. Significantly, the function of aforementioned proteins is usually affected by environmental toxicants which impair male fertility [8, 9]. Our results showed that OLA decreased the expression and localization of the junction proteins ZO-1, Occludin and N-cadherin at cell-cell interface. Mammalian target of rapamycin (mTOR) is a central regulator of cellular metabolic phenotype which integrates not only nutrient and energy-sensing pathways but also actin cytoskeleton organization [33, 34]. Herein, the steady-state protein level of rictor, a component of mTORC2 complex that is crucial for BTB dynamics [33, 37], was significantly decreased by OLA exposure, indicating that mTORC2 are potential mediators of OLA-induced BTB disruption. mTORC1/2 have been implicated in regulation of several testicular functions [36]. Depletion of mTOR in Sertoli cells impairs the organization of the seminiferous epithelium and the loss of cells polarity with a redistribution of BTB gap junction protein-1 (GJA1) [50]. In our study, the phosphorylation of mTOR^S2481^, p70 S6K^Thr389^ and Akt^Ser-473^ were reduced in OLA-exposed Sertoli cells, indicating that both mTORC1 and mTORC2 are molecular targets of OLA. Notably, mTORC1 and mTORC2 have been reported to have opposing effects on BTB dynamics as ribosomal protein S6 (rpS6, a pivotal downstream effector of mTORC1) compromises the permeability of BTB through down-regulating the expression of tight junction proteins whereas rictor, by orchestrating cytoskeleton architecture, promotes the BTB integrity [35, 37, 51]. Indeed, depletion of rictor was found to perturb the Sertoli cell TJ-barrier function and the BTB integrity due to the alteration of F-actin organization [37]. Herein, we also found that OLA could disrupt F-actin organization likely due to down-regulation of p-PKCα, which reminiscent of the finding that rictor knockdown leads to PKCα-mediated actin reorganization [52].

An overall reduction of F-actin bundles were found to across the entire cytosol with highly aggregated at the periphery of Sertoli cells. Actin regulator proteins such as small GTPases (e.g., Rac1 and CDC42) were strickly augmented. Moreover, CDC42 facilitates TGF-β3–mediated cascade of events that lead to the disruption of the TJ fibrils, which increases the endocytosis of occludin and ZO-1 at the BTB [53]. CDC42 accumulation promotes ZO-1 redistributed near the cell surface to cytosol [53]. In our study, the abundance of Arp2/3, which is a nucleation promoting factors and activated by ATP to induce branched actin nucleation [54], remains stable. However, the expression of N-WASP the activator of the Arp2/3 complex was drastically enhanced. Thus, we could not exclude the possibility that the Arp2/3 activity might be disrupted as the reduction in ATP content is induced in OLA-exposed Sertoli cells. Notably, palladin a actin-binding protein that promotes F-actin bundling also plays an important role in organizing actin filament arrays [55]. Both knockdown and overexpression of palladin result in the disorganization of actin cytoskeleton, and depletion of palladin leads to the loss of stress fibers and focal adhesions, and the cells become round up [56]. More importantly, palladin depletion also disrupts the permeability barrier integrity in Sertoli cell [31]. Collectively, these finding indicate that the F-actin disturbance induced by OLA exposure emerges as an important cue for the compromised TJ barrier permeability.

In mammalian cells, DNA damage was caused by intracellularly normal cellular metabolism, spontaneous mutations and external environment. Faithful maintenance and propagation of genetic and epigenetic information is essential for cell proliferation and survival [43]. Herein, we found that OLA-induced DNA damage which is presumably caused by ROS aggravation. The repair of DNA damage requires dynamic chromatin alterations comprising both transient decondensation and compaction of chromatin in the vicinity of DNA lesion sites [43, 57]. Double-strand break (DSB) repair initiates dynamic changes in histone modifications that are required to maintain genome stability [47, 58]. At DNA lesion sites, gene silencing is initiated by the recruitment of key proteins that involve in establishing and maintaining transcriptional repression, and conferring histone modifications including acetylation of H4K16 and methylation of H3K9me2/3 and H3K27me3 [59]. In higher eukaryotes, the methylation of H3K9 and H3K27 is linked to DNA replication and repair [47]. In our study, the global methylation of H3K9/H3K27 were significantly increased while the acetylation of H3K9/H3K27 were reduced after OLA-treatment (400 ug/ml), which sufficiently prove that methylation of H3K9 and H3K27 are linked to genotoxic stress in OLA-exposed Sertoli cells, implicating that the transient gene expression silencing is initiated until the completion of DNA repair is achieved.

Autophagy occurence in response to various stress stimulus, including oxidative stress, and DNA damage, which was considered to be a major protective mechanism against stress stimuli and plays an important role in many physiological processes [41, 60]. In this study, autophagic flux was inhibited while induced apoptosis in OLA-exposed Sertoli cells.Generally, autophagy block the induction of apoptosis, especially the stress is not lethal, while apoptosis-associated caspase activation shuts off the autophagic process when the intensity or duration of stress reaches the limit of the cell [61, 62]. Therefore, the aforementioned puzzle might be dispelled by the notion that the persistent DNA damage due to the failure in DNA repair revealed by the reduction in Rad51C expression predominately triggers apoptosis in the present OLA case in Sertoli cells. The accumulation of the autophagy receptor protein p62/SQSTM1 resulting from either inhibition or loss of autophagy can lead to impaired homologous recombination (HR)-mediated DNA damage repair [41, 63, 64]. Moreover, the up-regulation of SQSTM1/p62 also increases the ROS production [65]. In OLA-exposed Sertoli cells, the expression of SQSTM1/p62 is also increased, therefore, a possible mechanism could be inferred that the aggregation of ROS caused by SQSTM1/p62 up-regulation potentiates DNA damage. However, we could not exclude the possibility that a promiscuous interaction may exist among SQSTM1/p62, ROS and DNA damage as amelioration of ROS by NAC pretreatment could antagonize the accumulation of SQSTM1/p62 and DNA damage in OLA-exposed Sertoli cells. Autophagy is actived in Sertoli cells and plays an important role in maintain spermatogenesis [66, 67]. Sertoli cell-specific disruption of autophagy influences the fertility in male mouse caused by the resulting disorganized seminiferous tubules and abnormal spermatozoa with malformed heads due to disorganization of the cytoskeleton structures [67]. Thus, autophagy disruption might be another explanation for the disorganized cytoskeleton architecture induced by OLA exposure in Sertoli cells.

In conclusion, by deciphering that OLA perturbs TJ proteins cell-cell interface distribution and F-actin organization, which thereby compromises the BTB integrity and dynamics, we herein conceive that the Sertoli cells is sensitive to OLA-induced reproductive toxicity. Moreover, OLA could interferes with the epigenetic status of histone H3 presumably due to potentiated DNA damage, and the two main pathways apoptosis and autophagy that determine the cell fate in Sertoli cells. Our study provides the toxicological information of OLA in male fertility side, which might broaden our knowledge of the biological hazards of OLA to animal and our human health.

## MATERIALS AND METHODS

### Animals and ethnics statement

The present study was approved by the Ethical Committee of Hubei Research Center of Experimental Animals (Approval ID: SCXK (Hubei) 20080005). Wild-type Kunming (KM) mice were obtained from the local Central Animal Laboratory and housed in the experimental animal center of Huazhong Agricultural University under a 12 h light/12 h dark regimen at a temperature of 22°C with water and food ad libitum. All experimental procedures were performed in line with the guidelines of the Committee of Animal Research Institute, Huazhong Agricultural University, China.

### Chemicals and regents

Olaquindox (OLA, 2-(N-2’(hydroxymethyl) carboamoyl)-3-methylquinoxaline N1, N4-dioxide), C12H13N3O4, MW 263.25, CAS no. 23696-28-8, 99%) was purchased from Sigma-Aldrich (St. Louis, MO, USA) and prepared in dimethyl sulfoxide (DMSO) as a 400 mg/ml stock and diluted to its desired concentration with DMEM/F12 medium. The same amount of DMSO was used in control. N-acetylcysteine (NAC) was from Sigma-Aldrich (St. Louis, MO, USA), 10 mM of NAC was added 1 h before OLA treatment.

### Primary Sertoli cells isolation and treatment with OLA

Primary Sertoli cells were isolated from 3 weeks old male mice testes and cultured in serum-free DMEM/F12 medium supplemented with EGF (2.5 ng/ml), bovine insulin (10 μg/ml), human transferrin (5 μg/ml), bacitracin (5 μg/ml) and gentamicin (20 μg/ml) at 35°C with a humidified atmosphere of 5% CO_2_ in air as described [68]. Freshly isolated Sertoli cells were seeded on Matrigel-coated (Corning): (I) 6- or 12-well dishes at 0.5×10^6^ cells per cm^2^ were subsequently used for lysate preparation, (II) coated coverslips at 0.04-0.08×10^6^ cells per cm^2^ were processed to immunofluorescent analysis. The time of cell plating onto the dishes was defined as day 0. On day 2, cells were prior to hypotonic treatment using 20 mM Tris, pH 7.4, at 22°C for 2.5 minutes to lyse residual germ cells and then washed twice with DMEM/F12 medium. On day 3, OLA dissolved in DMSO was diluted in DMEM/F12 medium supplemented with various growth factors without bacitracin and gentamicin to obtain the desired final concentration. Sertoli cell were exposed to OLA for 24 h before termination. For the control, vehicle (i.e., DMEM/F12 medium containing DMSO at most 0.2% (V/V)) was used.

### Cell viability assay and morphology observation

Cell viability was detected by Cell Counting Kit-8 (CCK8) assays (Dojindo molecular technologies, Inc., Japan) according to the manufacturer’s instructions. Briefly, Sertoli cells were seeded in 96-well culture plates at a density of 0.5×10^5^ cells/well and treated with various concentrations of OLA (100-, 200-, 400- 800 μg/ml) for different length of time (24-48 h). Cell treated with vehicle (0.2 % DMSO) were used as a reference group with cell viability set as 100%. After treatments, the plate was carefully washed once in PBS at the indicated time, and then, 100 μl of medium containing CCK-8 solution was added to each well (the ratio of medium and CCK-8 volume was 9 : 1). After the plates were incubated for 1h at 37°C, the absorption at 450 nm of each well was measured using EnSpire® Multimode Reader (PerkinElmer, Inc., Waltham, USA). Cell used in this study were constantly observed under an inverted microscope (Nikon, Japan). Photographs were taken after Sertoli cells were treated with different concentrations of OLA for 24- and 48 h a described in the text.

### ATP assessment

ATP relative concentrations were evaluated by using the CellTiter-Glo® ATP Assay Kit (Promega Corp., Madison, WI, USA) according to the manufacturer’s instructions. Briefly, Sertoli cells were seeded in 96-well black culture plates at a density of 0.5×10^5^ cells/well and treated with various concentrations of OLA (100-, 200-, 400-, 800 μg/ml) for 24 h. After treatments, add a volume of CellTiter-Glo® Reagent equal to the volume of cell culture medium present in each well (add 100 μl of regent to 100 μl of medium containing cells for a 96-well plate) for 10 min at room temperature to stabilize luminescent signal. The plate was measured using EnSpire® Multimode Reader (PerkinElmer, Inc., Waltham, USA) to record luminescence. The data are shown as the average of five wells for each group.

### Caspase-3/7 activity assay

Caspase-3/7 activity was evaluated by using the Caspase-Glo® 3/7 Assay Kit (Promega Corp., Madison, WI, USA) according to the manufacturer’s instructions. Briefly, Sertoli cells were seeded in 96-well white culture plates at a density of 0.5×10^5^ cells/well and treated with various concentrations of OLA (100-, 200-, 400-, 800 μg/ml) for 24 h. Afterwards, add a volume of Caspase-Glo® 3/7 Assay Reagent equal to the volume of cell culture medium present in each well for 1 h at room temperature to stabilize luminescent signal. The plate was measured using EnSpire® Multimode Reader (PerkinElmer, Inc., Waltham, USA) to record luminescence. The data are shown as the average of five wells for each group.

### Functional assessment of the Sertoli cell TJ permeability barrier *in vitro* by trans-epithelial electrical resistance (TER) measurement

Primary Sertoli cell were plated on Millicell bicameral units (diameter, 12 mm; pore size, 0.45 μm, effective surface area, ∼ 0.6 cm^2^; Millipore) at 1.2×10^6^ cells per cm^2^ placed in 24-well dishes containing 0.5 ml F12/DMEM to monitored the Sertoli cell tight junction permeability barrier function by quantifying the trans-epithelial electrical resistance (TER) across the cell epithelium. On day 3, Sertoli cell were exposed to OLA (0, 100-, 200-, 400- and 800 μg/ml) for 24 h. Thereafter, cells were washed twice with F12/DMEM to remove chemicals reagents. The TER was assessed every 12 h until day 6 and each date was presented as mean ± S.E.M of n = 3 replicates as described. A total of four direction positions should be recorded for each bicameral culture unit. The true TER value was calculated as TERsample (Ωcm^2^) = (Resistancesample-Resistanceblank) (Ω) × Effective surface area (cm^2^) described by Mruk and Cheng [25].

### ROS determination

H_2_O_2_ was evaluated using ROS-Glo™ H_2_O_2_ Assay Kit (Promega Corp., Madison, WI, USA) according to the manufacturer’s protocol using a microplate reader. Sertoli cells plated at 1×10^5^ cells cells/well to a 96-well white plate and exposed to various concentrations of OLA (100-, 200-, 400-, 800 μg/ml) for 18 h before termination, 10 mM of NAC was added 1 h before OLA treatment. Less than 80μl of medium is desirable to accommodate addition of test compounds. Add 20 μl of H_2_O_2_ Substrate solution to cells and mix. The final well volume will be 100 μl, and the final H_2_O_2_ Substrate concentration will be 25 μM, incubate at 37°C in 5% CO_2_ for the final 6 h of treatment. Add 100 μl of ROS-Glo™ Detection Solution to each well and incubate for 20 mins at room temperature, and the luminescence was determined with EnSpire ® Multimode Reader (PerkinElmer, Inc., Waltham, USA).

### Immunofluorescence

For immunofluorescent analysis, Sertoli cells cultured at 0.04 × 10^6^ cells per cm^2^ on Matrigel-coated coverslips were fixed with 4% paraformaldehyde (wt/vol) in PBS for 10 min, permeabilized in 0.1% Triton X-100/PBS (vol/vol) for 4 mins. Cells were then blocked with 5% BSA/PBS (wt/vol) for 1 h, followed by an overnight incubation of primary antibodies. After washing in PBS/0.1%Tween/0.01%Triton X-100, cells were processed to incubation with corresponding secondary antibodies at 4°C for 16 h and 37°C for 2 h, respectively. For F-actin staining, Sertoli cells were incubated with FITC-conjugated phalloidin (Sigma, MO). DNA was visualized with DAPI (10 μg/ml) for 15 mins at room temperature and then coverslips were mounted to slides with DABCO followed by examination with confocal laser scanning microscope (ZEISS LSM 510 META, Carl Zeiss Imaging, Germany) equipped with a Plan-Apochromat 40 ×/1.4 oil DIC objective. Confocal images were processed using Zeiss LSM Image Browser software and Adobe Photoshop (Adobe Systems Inc., San Jose, CA). For the negative control, non-immunized rabbit or goat IgG were used to replace the primary antibodies. Antibodies and dilutions were listed in Table 1.

**Table 1:** The information of antibodies used in IF and WB analyses

Product	Catalog	Application and dilution	
		WB	IF
ZO-1 antibody	61-7300, Invitrogen	1:100	1:50
occludin antibody	71-1500, Invitrogen	1:100	
N-cadherin antibody	Ab76011, Abcam	1:1000	
	14215, CST		1:100
Phospho-Connexin 43 antibody	3511, CST	1:1000	
FAK	AF6397, Affinity	1:500	
Phospho-FAK	AF3398, Affinity	1:500	
Phospho-P38 MAPK antibody	4511, CST	1:1000	
Phalloidin-FITC	P5282, Sigma		1:100
Rac1antibody	2465, CST	1:1000	
CDC42 antibody	ab187643, Abcam	1:1000	
N-WASP antibody	ab126626, Abcam	1:1000	
Arp3 antibody	ab181164, Abcam	1:2000	
Palladin	DF9731, Affinity	1:500	
RICTOR	DF7530, Affinity	1:500	
Phospho-mTOR antibody	DF3308, Affinity	1:500	
Akt antibody	AF6261, Affinity	1:500	
Phospho-Akt antibody	7160, CST	1:1000	
Phospho-p70 S6 Kinase antibody	AF3228, Affinity	1:500	
PKCαβ antibody	ab179522, Abcam	1:1000	
Phospho-PKC antibody	AF8396, Affinity	1:500	
Cleaved-PARP antibody	AF7023, Affinity	1:500	
Cleaved-Caspase 3 antibody	AF7022, Affinity	1:500	
Bax antibody	AF0083, Affinity	1:500	
Beclin1 antibody	AF5128, Affinity	1:500	
P62/SQSTM1 antibody	AF5384, Affinity	1:1000	
LC3A/B antibody	1274, CST	1:1000	
Phospho-H2A.X antibody	9718, CST	1:1000	1:100
Rad51C antibody	NB100-177, Novus	1:1000	
H3K9ac antibody	Ab10182, Abcam	1:1000	
H3K27ac antibody	39134, Active Motif	1:1000	
H3K9me1 antibody	DF6936, Affinity	1:500	
H3K9me2 antibody	DF6937, Affinity	1:500	
H3K9me3 antibody	39285, Active Motif	1:500	
H3K27me1 antibody	DF6939, Affinity	1:500	
H3K27me2 antibody	DF6940, Affinity	1:500	
H3K27me3 antibody	39155, Active Motif	1:1000	
β-Actin antibody	BF0198, Affinity	1:2000	
HRP-GAPDH antibody	AB2000, Abways	1:5000	
HRP-goat anti-rabbit antibody	sc2004, Santa Cruz	1:3000	
HRP-goat anti-mouse antibody	sc-2005, Santa Cruz	1:3000	
Cy3-goat anti-rabbit antibody	111-166-045, Jackson		1:100

### Immunoblot assay

Sertoli cell washed with ice-cold PBS, and lysed in extraction buffer (50 mM Tris, 150 mM NaCl, 1% Triton X-100, 1% sodium deoxycholate, 0.1% SDS, pH 7.4) supplemented with protease and phosphatase inhibitor mixtures and PMSF (Sigma, MO), sonicating, and centrifuging to obtain the clear supernatant. Lysates were stored at -80°C until used. For immunoblot analysis, lysates (30 μg of protein) were separated by SDS-polyacrylamide gel, blotted onto PVDF membrane (Immobilon-P; Millipore), and blocked in TBS/0.1% Tween-20/5% BSA (TBS, 25 mM Tris, 150 mM NaCl, pH 7.4) for 1 h, followed by primary antibodies incubation for overnight at 4°C. After washing in TBS/0.1% Tween-20, membranes were subjected to incubation with corresponding HRP-conjugated secondary antibodies. The immunoblot bands were visualized with ECL kit and read using chemiluminescence system (Thermo Scientific, Waltham, MA). Antibodies and dilutions were listed in Table 1.

### Statistical analysis

Data from at least 3 replicates was presented as mean ± S.E.M. Comparisons of two datasets were performed using unpaired two-tailed Student’s *t*-test. All other comparisons of multiple data-sets were performed using one-way ANOVA followed by Tukey’s *post hoc* test with p < 0.05 was considered to be statistically significant.

## SUPPLEMENTARY MATERIALS FIGURE



## Abbreviations

OLA: olaquindox
NAC: N-acetylcysteine
BTB: blood-testis barrier
TJ: tight junction
basal ES: basal ectoplasmic specializations
GJ: gap junction
ROS: reactive oxygen species
TER: trans-epithelial electrical resistance
