# Supplementary material for: Olaquindox disrupts tight junction integrity and cytoskeleton architecture in mouse Sertoli cells
Source: Oncotarget. 2017 Aug 16;8(51):88630–44. doi: 10.18632/oncotarget.20289 (PMC5687633; doi:10.18632/oncotarget.20289)
Supplement: Supplementary file 1 [file oncotarget-08-88630-s001.pdf]

Olaquindox disrupts tight junction integrity and cytoskeleton architecture in mouse Sertoli cells

SUPPLEMENTARY MATERIALS

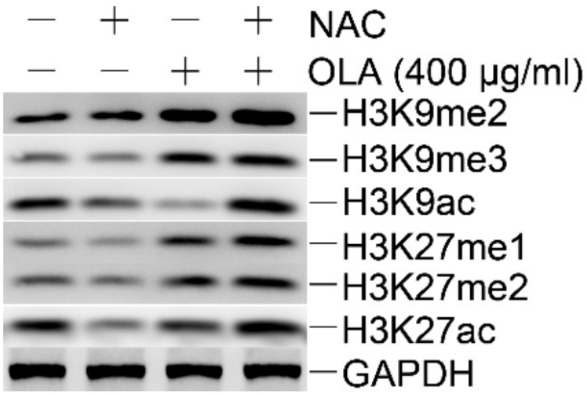

Supplementary Figure 1: NAC rescued hypoacetylation of H3K9 and H3K27 induced by OLA. Sertoli cells were pretreated with NAC (10 mM) for 1 h, followed by 400 μg/ml OLA treatment for 24 h prior to immunblot analysis for the expression of H3K9 and H3K27 modification. GAPDH served as protein loading control.
